# Regenerative Effect of Growth Hormone (GH) in the Retina after Kainic Acid Excitotoxic Damage

**DOI:** 10.3390/ijms20184433

**Published:** 2019-09-10

**Authors:** Carlos G. Martinez-Moreno, David Epardo, Jerusa E. Balderas-Márquez, Thomas Fleming, Martha Carranza, Maricela Luna, Steve Harvey, Carlos Arámburo

**Affiliations:** 1 Departamento de Neurobiología Celular y Molecular, Instituto de Neurobiología, Campus Juriquilla, Universidad Nacional Autónoma de México, Querétaro, Qro., 76230, Mexico (D.E) (J.E.B.-M.) (T.F.) (M.C.) (M.L.); 2 Department of Physiology, University of Alberta, Edmonton, AB T6G 2H7, Canada

**Keywords:** growth hormone, retina, regeneration, synaptogenic, neurotrophic, excitotoxicity

## Abstract

In addition to its role as an endocrine messenger, growth hormone (GH) also acts as a neurotrophic factor in the central nervous system (CNS), whose effects are involved in neuroprotection, axonal growth, and synaptogenic modulation. An increasing amount of clinical evidence shows a beneficial effect of GH treatment in patients with brain trauma, stroke, spinal cord injury, impaired cognitive function, and neurodegenerative processes. In response to injury, Müller cells transdifferentiate into neural progenitors and proliferate, which constitutes an early regenerative process in the chicken retina. In this work, we studied the long-term protective effect of GH after causing severe excitotoxic damage in the retina. Thus, an acute neural injury was induced via the intravitreal injection of kainic acid (KA, 20 µg), which was followed by chronic administration of GH (10 injections [300 ng] over 21 days). Damage provoked a severe disruption of several retinal layers. However, in KA-damaged retinas treated with GH, we observed a significant restoration of the inner plexiform layer (IPL, 2.4-fold) and inner nuclear layer (INL, 1.5-fold) thickness and a general improvement of the retinal structure. In addition, we also observed an increase in the expression of several genes involved in important regenerative pathways, including: synaptogenic markers (DLG1, NRXN1, GAP43); glutamate receptor subunits (NR1 and GRIK4); pro-survival factors (BDNF, Bcl-2 and TNF-R2); and Notch signaling proteins (Notch1 and Hes5). Interestingly, Müller cell transdifferentiation markers (Sox2 and FGF2) were upregulated by this long-term chronic GH treatment. These results are consistent with a significant increase in the number of BrdU-positive cells observed in the KA-damaged retina, which was induced by GH administration. Our data suggest that GH is able to facilitate the early proliferative response of the injured retina and enhance the regeneration of neurite interconnections.

## 1. Introduction

Neurotrophic factors, tissue organization, and microenvironmental dynamics are key elements to understand adult neurogenesis and neuroregeneration [1,2]. Cell interactions and crosstalk between neurons, glial cells, and microvasculature in neural tissue can induce or repress the renewal of progenitors, cell transdifferentiation, axonal re-growth, and synaptic plasticity [3,4,5]. Considering the increasing number of reports regarding the neuroprotective effects of GH in several models as well as the beneficial impacts of GH treatment in patients with brain trauma, stroke, spinal cord injury, neurological deficit, and cognitive disfunction, it is necessary to fully understand the molecular mechanisms and cellular interactions underlying these neurotrophic effects [6,7,8,9].

A common ground in vertebrate species is the potential capacity of organisms to induce retinal neuroregeneration after injury. It is known, however, that while cold-blooded vertebrates retain a robust capacity for neuroregeneration into adulthood [10,11], the regenerative potential of avian and mammalian models is absent or largely restricted to the early stages of development [12,13]. Müller cells, the retinal pigment epithelium, and the ciliary marginal zone are a common potential source of neural progenitors in the retina, though the efficiency and regulation of these neurogenic niches—cellular interactions, signaling pathways, and local factors—may differ among species [10,11]. In particular, the chicken retina shows a well-studied regenerative response that involves Müller cell transdifferentiation, which can be induced by the administration of an excitotoxic agent during the first week of post-hatch development [10]. However, neuroregenerative process does not restore neural function through cell replacement and functional neurogenesis has not been described yet [10,14]. Nevertheless, this model allows for the study of the signals that trigger Müller cell de-differentiation and the factors which may prevent functional recovery in response to injury [15].

Avian retinal cells are the target of GH stimulation since the GH receptor (GHR) expression has been described there, particularly in neuroretinal and glial cells, in which axogenic and synaptogenic effects are well established [16,17,18,19,20]. It is also important to note that both GH and IGF-1 are neuroprotective factors that are also expressed in the retina [17], and their actions are mediated through signaling pathways such as JAK/STAT, IP3/Akt, MAPK, and Notch, which are activated during the neuroregenerative process [7,21,22,23].

This study shows that, after damage by KA-treatment, a chronic GH administration protocol in the retina partially restored the structure of several retinal cell layers and significantly increased the expression of several factors involved in regeneration, whose characteristics are briefly described in Table 1. These include various axonal/synaptic markers (GAP43, NRXN1, SNAP25, and DGL1), glutamate receptor subunits (NR1 and GRIK4) and Notch signaling elements (Notch1 and Hes5). Neurotrophic actions of GH in the neural retina were also evaluated through changes in the expression of FGF2, BDNF, BMP4, Bcl-2, and TNF-R2. Proliferative actions of GH were evaluated through BrdU (short-term) and PCNA (long-term) assays. These results suggest that GH enhances partial neuroregeneration after a strong and acute excitotoxic injury induced by KA, particularly by increasing the molecular markers of neural interconnections and the promotion of neural stemness.

## 2. Materials and Methods

### 2.1. Animals

Fertilized eggs (*Gallus gallus*, White Leghorn) were kindly donated by Pilgrim’s Pride (Querétaro, México) and were incubated at 39 °C in a humidified air chamber (IAMEX, México). The eggs were rotated one-quarter of a revolution every 50 min until hatch. One-day old chickens were used to start chronic treatments and after 3 weeks they were sacrificed. Animals were anesthetized with subcutaneous xylazine (1 mg/kg) and ketamine (2.2 mg/kg) prior to intravitreal injections. After treatments, the chickens were sacrificed by decapitation following a protocol approved by the Institute’s Bioethics Committee (Instituto de Neurobiología, UNAM).

### 2.2. Administration of Intraocular Injections

KA (20 µg/dose) (Cat. K2389; Sigma Aldrich, St Louis, USA) and/or recombinant chicken GH (rcGH; 300 ng/dose) (Revholt, PRL, Israel) were suspended in 10 µl of sterile saline solution. Intravitreal injections with ultra-fine syringes (gauge needle 31G) were applied into the left eyes for treatments, while vehicle was injected in the contralateral right eyes used as sham controls [7].

**Figure 1:**
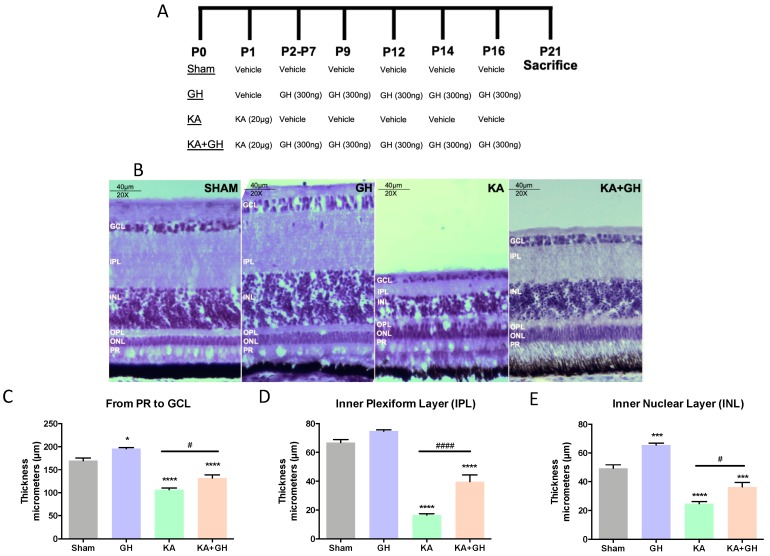
Morphometric analysis showing the regenerative effect of growth hormone (GH) after kainic acid (KA)-induced excitotoxicity in chicken retinas. (**A**) Treatments and time-line schematic representation of intravitreal injection protocols in the experimental groups. Units in nanograms (ng) and micrograms (μg). P: postnatal day. (**B**) Histological analysis in hematoxylin-stained chicken retinal slices treated with either KA (damage), GH (treatment) or GH + KA (neuroprotection treatment) in the left eye. Sham (vehicle injected) as negative control (right eye). The thickness of retinal layers was quantified after each treatment: (**C**) from photoreceptors (PR) to ganglion cell layer (GCL); (**D**) inner plexiform layer (IPL) and (**E**) inner nuclear layer (INL). Bars indicate thickness mean ± SEM (*n* = 3–5 animals per group, 3 fields were quantified per retina/animal). Asterisks indicate significant difference in comparison to control (*, *p* < 0.05; ***, *p* < 0.001, ****, *p* < 0.0001) and number sign (#) shows difference between experimental groups (#, *p* < 0.05; ####, *p* < 0.0001) as determined by one-way ANOVA for multiple comparisons and Šidák as *post-hoc* test.

The regenerative effect of GH was determined over a 3-week long administration protocol that included a single injury shot with KA and 10 serial GH injections starting 1 day after the excitotoxic insult (Figure 1A). Intravitreal injections were administered into the left eye as follows: injection of KA, 6 daily injections of GH (stage 1), and 4 injections with a 2-day delay between administrations (stage 2). Once administration stages 1 and 2 were finished, retinas were collected 5 days after the last GH injection (at day 21 post-injury) in order to avoid short-term and transient effects of the treatment.

To evaluate the effect of treatments upon retinal cell proliferation, a BrdU labeling assay was employed. KA was intravitreally injected at postnatal day 1 (P1) followed by a 6-day GH administration scheme (Figure 5A). Eyes were collected and fixed at P8 (7 days after damage), following sacrifice. Sham controls were included. We based our multi-dose experimental designs on Fischer and Reh [56,57] reports, in which serial doses of growth factors induced neurogenesis and neuroregeneration.

### 2.3. Quantification of Gene Expression by Quantitative Reverse Transcription Polymerase Chain Reaction (RT-qPCR)

Total RNA was purified from retinal lysates using TRIzol (Zymo Research Corp. Irvine, CA, USA) and Zymo Direct-zol purification kit. Genomic DNA was digested with DNAse I for 15 min at room temperature (RT) [7]. cDNA was synthesized from 1.0 μg of total RNA using oligo (dT) and random hexamers. Retrotranscription was performed with 100 U of M-MLV reverse transcriptase (Promega, Madison, WI, USA) and 1 mM dNTPs for 60 min at 42 °C. The expression of target genes (Table 2) were quantified by real time PCR (RT-qPCR) in an ABI-PRISM 7900HT sequence detection system (Applied Biosystems, Foster, CA, USA), using SYBR Green (Maxima, Thermo Scientific, Waltham, MA, USA) in 10 µl final volume containing: 3 µL of diluted cDNA and 0.5 mM of each specific primer. Reactions were performed as follows: initial denaturation at 95 °C for 10 min, then 45 cycles of 95 °C for 15 s, 60 °C for 15 s and 72 °C for 15 s. Relative abundance of mRNA was calculated using the comparative threshold cycle (Ct) method and employing the formula 2^−∆∆CT^ [58] where the quantification is expressed relative to the geometric mean of 18S mRNA [59,60,61,62]. Gene expression determinations were performed in duplicates from 4–5 animals per experimental group.

### 2.4. Western Blot Analysis

Western blot analysis was performed as previously reported [7,19,20,21]. Total proteins were extracted from retinas using a homogenization buffer (HCl-Tris 0.05 M, pH 9.0) containing a cocktail of protease inhibitors (Mini-complete, Roche, Mannheim, Germany). After centrifugation (12500 rpm, 10 min), a similar amount of protein from each sample supernatant (60 μg, as determined by the Bradford assay) was loaded and separated in 12.5% sodium dodecyl sulfate polyacrylamide gel electrophoresis (SDS-PAGE) and transferred onto nitrocellulose membranes (Bio-Rad, Hercules, CA, USA). Nitrocellulose-free binding sites were blocked with 5% non-fat milk (Bio-Rad, Cat.No.170–6404, Hercules, CA, USA) in Tris-buffered saline (TBS) for 2 h at room temperature. Membranes with protein samples were incubated overnight at RT with the corresponding primary antibody (Table 3) in TTBS (1 × TBS with 0.05% tween [v/v]). After washing the membranes with TTBS (2 × 5 min), they were then incubated for 2 h with the corresponding horseradish peroxidase (HRP) conjugated secondary antibody. Bands were visualized using ECL Blotting Detection Reagent (Amersham-Pharmacia, Buckinghamshire, UK) on autoradiography film (Fujifilm, Tokyo, Japan). Kaleidoscope molecular weight markers (Bio-Rad) were used as a reference for apparent molecular weight determination. Images were captured on Gel Doc EZ Imager (Bio-Rad) and immunoreactive bands were analyzed by densitometry using Image Lab Software (Bio-Rad). Target immunoreactivities were corrected using β-actin as loading control. Western blot analysis included 6–10 animals per experimental condition.

### 2.5. Histochemistry and Bromodeoxyuridine (BrdU) Assay

Enucleated eyes were hemisected longitudinally and the vitreous was removed. Eyes were fixed in 4% paraformaldehyde + 3% sucrose in PBS for 1 h. Samples were washed with PBS (3 × 5 min), cryoprotected in 30% sucrose, and freeze mounted onto aluminum sectioning blocks with Tissue-Tek^®^ O.C.T (Sakura Finetek, Torrance, CA, USA). Sections of 15 µm were cut with a cryostat (Leica CM3050 S, Bufalo Grove, IL, USA) and then mounted on pretreated glass slides (Fisherbrand, Fisher Scientific, Waltham, MA, USA). For histological analysis, slides were stained with hematoxylin [7]. Retinal cell-layer thickness was determined in slices stained with hematoxylin in at least 3 microscopic fields per eye, and 3–5 individual eyes per experimental group were examined. Retinal morphometrical analysis was performed in the same area for equivalent group comparisons. The criteria of ~1–3 mm from the head of the optic nerve was applied for image capturing.

To determine cell proliferation, BrdU was administered via intravitreal (20 µg at day P5) and intraperitoneal (1.5 mg at day P6) injections following the protocol shown in Figure 5A [48]. BrdU incorporation into the cell nuclei was determined by immunohistochemistry. Briefly, retinal slides were washed with TBS (3 times for 10 sec) and for DNA hydrolysis were incubated with HCl (2M) for 20 min. After hydrolysis, the tissue slides were washed with TBS (3 times for 10 sec). For immunohistochemical analysis, BrdU was determined with a rat monoclonal anti-BrdU antibody (Abcam, Cambridge, UK) diluted 1:100 in PBS plus 0.2% Triton X-100. Secondary goat anti-rat IgG antibody was diluted 1:2000 in TPBS with 1% non-fat dry milk (Bio-Rad) and incubated for 2 h at RT. Controls without primary antibodies were included. Sections were stained with DAPI. Images were captured using an Olympus BX51 fluorescence microscope (Tokyo, Japan) and analyzed with Image Pro software (Media Cybernetics, Rockville, MD, USA). BrdU positive cells were determined in 3 fields per animal from 3–5 animals.

### 2.6. Statistical Analysis

Each experimental condition (left eye) used its contralateral eye (right; sham control) as a reference for relative change (delta). In all the experiments, values are expressed as mean ± SEM. Significant differences between groups or treatments were determined by one-way ANOVA analysis followed by Šidák’s *post-hoc* test. P-values less than 0.05 were determined to be statistically different (*, *p* < 0.05; **, *p* < 0.01; ***, *p* < 0.001; **** *p* < 0.0001).

## 3. Results

### 3.1. Effect of Growth Hormone (GH) on Neuroregeneration in KA Damaged Retinas

Retinas were damaged by the in vivo application of a single dose of KA (20 µg) and treated post-injury with 10 intravitreal injections of GH (300 ng/dose) over 3 weeks, following the protocol depicted in Figure 1A. Histological analysis of the retinal tissues stained with hematoxylin revealed that, in comparison to the control, important morphological changes occurred in all treatment groups; however, the most evident cytostructural modification occurred in KA-damaged groups where a severe disruption of cellular organization in several retinal layers was observed (Figure 1B). Interestingly, a significant structural restoration of layers was found in the KA + GH group. Morphometric analysis, performed by measuring changes in layer thickness, showed that KA caused a significant decrease in retinal thickness (from photoreceptors (PR) layer to ganglion cell layer (GCL) (63.1 ± 3.4 µm; *p* < 0.0001), as well as a drastic reduction in the thickness of the inner plexiform layer (IPL) (51.6 ± 2.01 µm; *p* < 0.0001) and the inner nuclear layer (INL) (29.5 ± 1.6 µm; *p* < 0.0001) (Figure 1C–E), when compared to their respective controls. Conversely, the thickness of IPL and INL was significantly increased (25.4 ± 3.1 µm and 14.6 ± 3.6 µm; *p* < 0.001 and *p* < 0.05, respectively) in the KA + GH group, in comparison to the KA-damaged group. On the other hand, the administration of GH alone promoted a significant increase in retinal thickness (25.9 ± 3.1 µm; *p* < 0.05) (Figure 1C) and INL thickness (10.9 ± 2.6 µm; *p* < 0.001) (Figure 1E), with respect to the control.

### 3.2. Growth Hormone (GH) increases Synaptogenic Markers and Glutamate Receptor Expression in Response to Injury

**Figure 2:**
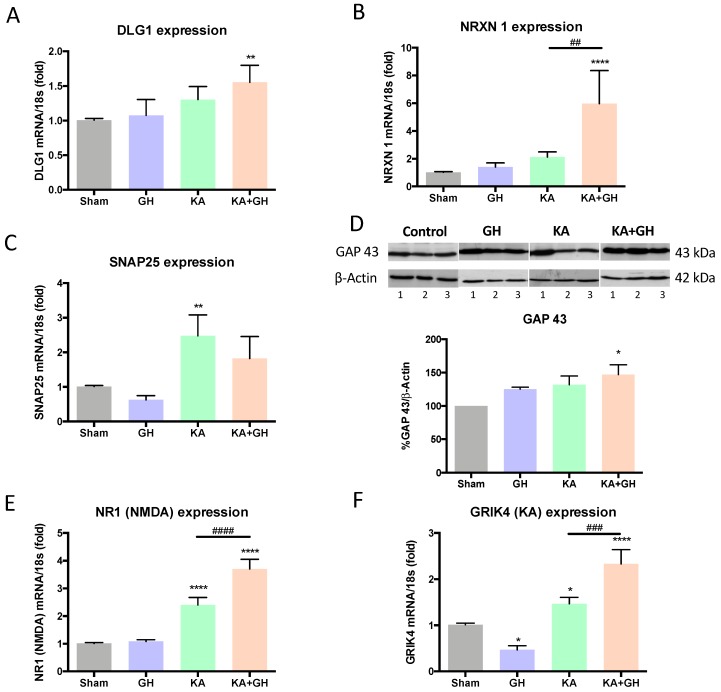
Effects of growth hormone (GH) upon DLG1, NRXN1, SNAP25, NR1, and GRIK mRNAs expression, and GAP43 immunoreactivity in chicken retinas exposed to excitotoxic damage. Panels show the relative expression of: (**A**) DLG1, (**B**) NRXN1, (**C**) SNAP25, (**E**) NR1, and (**F**) GRIK4 mRNAs, as determined by RT-qPCR. Relative mRNA expression values were corrected by the comparative threshold cycle (Ct) method and employing the formula 2^−∆∆CT^. Ribosomal 18S RNA was used as housekeeping gene. (**D**) GAP43 immunoreactivity was analyzed by western blot and densitometry and normalized using β-actin as a loading control; 1, 2 and 3 represent different samples. A representative luminogram is shown. Bars represent mean ± SEM (*n* = 4–5; analyzed by duplicate). Asterisks indicate significant difference in comparison to control (*, *p* < 0.05; **, *p* < 0.01; ****, *p* < 0.0001) and number sign (#) shows difference between experimental groups (##, *p* < 0.01; ###, *p* < 0.001; ####, *p* < 0.0001) as determined by one-way ANOVA for multiple comparisons and Šidák as *post-hoc* test.

The expression of several synaptogenic markers in response to KA-injury in the treated retinas was analyzed by either RT-qPCR or western blot (Figure 2). It was found that DLG1 (a post-synaptic element [20]) mRNA expression was significantly increased (0.5-fold; *p* < 0.01) in the KA + GH experimental group in comparison to the sham group (Figure 2A), while that of neurexin-1 (a presynaptic protein, NRXN1) was strongly upregulated in the KA + GH group in comparison to sham (4.8-fold; *p* < 0.0001), GH (3.2-fold; *p* < 0.0001), and KA groups (2.9-fold; *p* < 0.01) (Figure 2B). In turn, SNAP25 (a post-synaptic component) mRNA expression levels showed a significant increase (*p* < 0.01) only in the KA damaged group in relation to control (1.4-fold; *p* < 0.01) and GH (2.9-fold; *p* < 0.01) groups (Figure 2C). On the other hand, the immunoreactivity of the axonal and neurite growth marker GAP43 also increased (43%; *p* < 0.05) in response to GH after KA-damage (Figure 2D).

KA-induced excitotoxicity significantly increased NR1 and GRIK4 mRNA expression levels (1.3-fold and 0.4-fold; *p* < 0.0001 and *p* < 0.05, respectively) in comparison to the control group. The administration of GH after damage (KA + GH) further enhanced the expression levels of both glutamate receptor subunits over the KA-injured group (0.6-fold and 0.5-fold; *p* < 0.0001 & *p* < 0.001, respectively) (Figure 2E,F). In retinas treated with GH alone, GRIK4 mRNA expression showed a significant decrease (0.6-fold; *p* < 0.05) in comparison to the control.

### 3.3. Pro-survival Effects of Growth Hormone (GH) in the Damaged Retina

**Figure 3:**
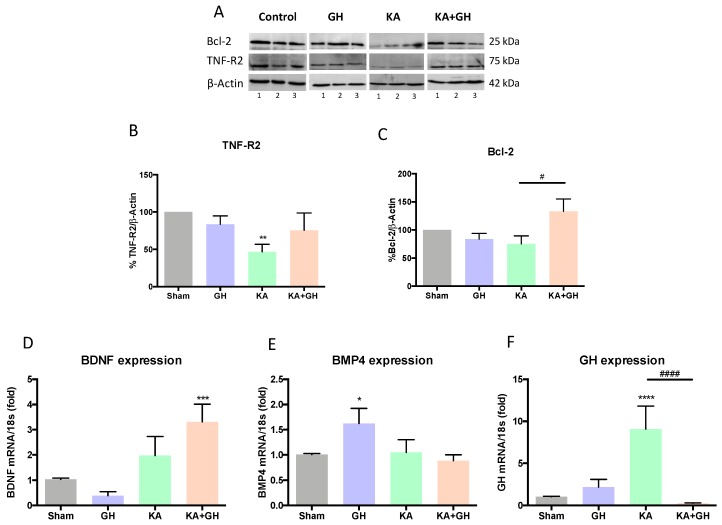
Effect of growth hormone (GH) upon Bcl-2 and TNF R2 immunoreactivity and BDNF, BMP4 and GH mRNAs expression in chicken retinas exposed to excitotoxic damage.(**A**) Representative western blot luminograms for Bcl-2 and TNF-R2 immunoreactivities in experimental groups. β-Actin was included as a loading and normalizing control. 1, 2 and 3 represent different samples. Immunoreactive bands were analyzed by densitometry for TNF-R2 (**B**) and Bcl-2 (**C**). Relative expression of: (**D**) BDNF, (**E**) BMP4, and (**F**) GH mRNAs, as determined by RT-qPCR. Relative mRNA expression values were corrected by the comparative threshold cycle (Ct) method and employing the formula 2^−∆∆CT^. Ribosomal 18S RNA was used as housekeeping gene. Bars represent mean ± SEM (*n* = 4; analyzed by duplicate). Asterisks indicate significant difference in comparison to control (*, *p* < 0.05; **, *p* < 0.01; ***, *p* < 0.001; ****, *p* < 0.0001) and number sign (#) shows difference between experimental groups (#, *p* < 0.05; ####, *p* < 0.0001) as determined by one-way ANOVA for multiple comparisons and Šidák as *post-hoc* test.

The relationship between the neuroprotective effect of GH and other cell survival factors was also explored (Figure 3). It is known that the pro-survival actions of TNFα are mediated by TNF-R2. Results showed that TNF-R2 immunoreactivity was significantly decreased (52%; *p* < 0.01) in retinas injured by KA, but it was restored to control levels in the KA + GH group (Figure 3A,B). Also, Bcl-2 immunoreactivity was significantly increased (58%; *p* < 0.05) in the KA + GH group in comparison to the KA group (Figure 3C). Likewise, co-treatment with KA + GH significantly increased BDNF mRNA expression in comparison to the control group (2.2-fold; *p* < 0.001) (Figure 3D). On the other hand, BMP4 mRNA expression increased significantly only in response to GH treatment alone (0.6-fold; *p* < 0.05); in this case, however, GH did not exert any effect in response to damage (Figure 3E). Finally, in KA-damaged retinas, locally expressed GH mRNA was significantly increased (7.7-fold; *p* < 0.0001) and this response was totally abrogated in the KA + GH group (Figure 3F).

### 3.4. Effect of Growth Hormone (GH) Treatment on Cell Transdifferentiation and Notch Signaling Pathway

**Figure 4:**
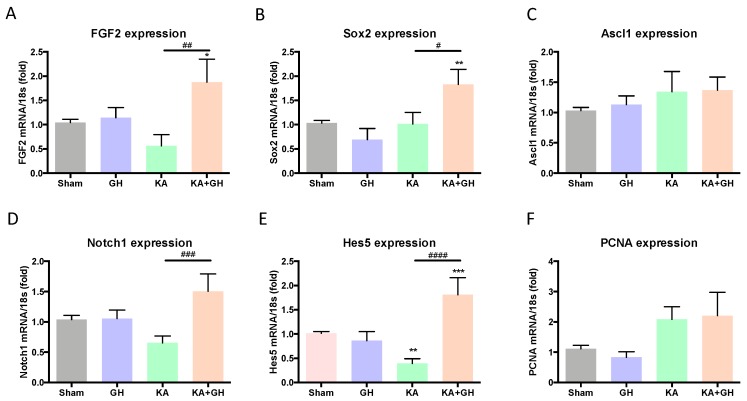
Effect of growth hormone (GH) upon FGF2, Sox2, Ascl1, Notch1, Hes5 and PCNA mRNAs expression in chicken neuroretinas exposed to excitotoxic damage. Panels show the relative expression of (**A**) FGF2, (**B**) Sox2, (**C**) Acsl1, (**D**) Notch1, (**E**) Hes5, and (**F**) PCNA mRNAs, as determined by RT-qPCR. Relative mRNA expression values were corrected by the comparative threshold cycle (Ct) method and employing the formula 2^−∆∆CT^. Ribosomal 18S RNA was used as housekeeping gene. Bars represent mean ± SEM (*n* = 4–5, analyzed by duplicate). Asterisks indicate significant difference in comparison to control (*, *p* < 0.05; **, *p* < 0.01; ***, *p* < 0.001) and number sign (#) shows difference between experimental groups (#, *p* < 0.05; ##, *p* < 0.01; ###, *p* < 0.001; ####, *p* < 0.0001) as determined by one-way ANOVA for multiple comparisons and Šidák as *post-hoc* test.

Trans-differentiation cell markers were analyzed to evaluate GH actions during the retinal regenerative process that occurs after excitotoxic injury (Figure 4). Expression of FGF2 and Sox2 mRNAs significantly increased (0.7-fold and 0.8-fold; *p* < 0.05 and *p* < 0.01, respectively) in the KA + GH group in comparison to KA-damaged group (Figure 4A,B). Independent GH or KA treatments were not different from the control. The transcription factor Ascl1 mRNA expression, however, showed no significant difference in any experimental group (Figure 4C).

Notch signaling activation was determined by Notch1 and Hes5 mRNAs expression. Levels of Notch1 mRNA showed a significant increase (1.2-fold; *p* < 0.001) in the group treated with KA + GH in comparison to the KA-damaged group (Figure 4D). On the other hand, KA treatment significantly decreased the expression of Hes5 mRNA (0.62-fold; *p* < 0.01) compared to the control, while post-injury GH administration provoked a drastic increase in Hes5 mRNA levels over sham, GH, and KA groups (0.7-fold and 3.6-fold; *p* < 0.001 and *p* < 0.0001, respectively) (Figure 4E). Both Ascl1a and PCNA mRNA expression did not show any significant differences between groups (Figure 4C,F).

### 3.5. Proliferative Effect of Growth Hormone (GH) in the Damaged Retina

**Figure 5:**
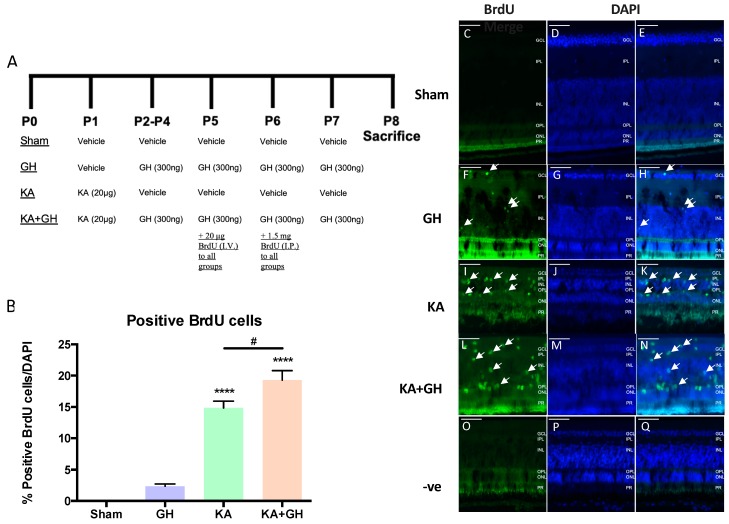
Proliferative effect of growth hormone (GH) treatment over BrdU immunoreactivity in the chicken retina exposed to excitotoxic damage. (**A**) Treatments and time-line schematic representation of intravitreal injection protocols in the experimental groups. Units in nanograms (ng), micrograms (μg) and milligrams (mg). P: postnatal day, I.V. intravitreal, I.P. intraperitoneal. (**B**) Positive BrdU cells, quantified as relative percentage in relation to DAPI labeled cells. BrdU immunofluorescence in control (sham), GH, kainic acid (KA) and KA + GH treated retinas. Sham (vehicle) and treated retinas were stained with DAPI (Blue; D, G, J, M, P), and with specific antibodies directed against BrdU (Green; C, F, I, L, O). Merged images are shown in panels E, H, K, N, and Q. Negative controls without primary antibody (O, P, and Q). Arrows denote co-localization of BrdU and DAPI in the same cells. Bars represent mean ± SEM (*n* = 3–5 animals per group, 3 fields were quantified per retina/animal). Asterisks indicate significant difference in comparison to control (****, *P* < 0.0001) and number sign (#) shows difference between experimental groups (#, *P* < 0.05) as determined by one-way ANOVA for multiple comparisons and LSD Fischer as *post-hoc* test.

The proliferative effect of GH after KA treatment was determined by BrdU cell labeling in retinal slices (Figure 5). Arrows show examples of BrdU positive cells (Figure 5C–N). One week after damage (Figure 5A), we observed a significant increase in the number of BrdU-positive cells in both the KA-damaged (13-fold; *p* < 0.0001) (Figure 5B,I–K) and the KA + GH groups (18-fold; *p* < 0.0001) (Figure 5B,L–N) in comparison to control retinas, which showed no BrdU staining (Figure 5B–E). Negative controls without primary antibody did not show BrdU-positive cells (Figure 5O,Q). Cellular proliferation induced by KA treatment was further enhanced by GH co-administration (0.3-fold; *p* < 0.05) (Figure 5B). Although 2–3 % of cells in GH treated retinas were BrdU positive, it was not statically different from the control group (when analyzed by one-way ANOVA) (Figure 5B).

## 4. Discussion

This work aimed to study the involvement of GH in the regenerative response observed in the avian retina exposed to excitotoxic damage. To this end, we evaluated the expression of several axonal/synaptic markers, glutamate receptor subunits, transcription factors, and Notch signaling elements known to participate in the generation of retinal neuronal precursors derived from Müller cells, after provoking a damage with KA and the effect of long term (3 weeks) GH administration post-injury. Our results indicate, for the first time, an enhancing effect of GH upon avian retinal neuroregeneration.

A single excitotoxic injury in the chicken retina administered at postnatal day 1 is capable to induce Müller cell transdifferentiation; this process includes two stages: (a) cell de-differentiation and (b) cell proliferation [14]. The proliferative stage is significantly decreased after 14 days post-injury [63]. Our protocol included two GH administration stages (300 ng/dose): an early stage including 6 daily doses followed by a second stage with 4 doses every third day in order to prevent GHR desensitization.

We previously demonstrated that a preventive/protective GH treatment applied 1 h before and simultaneously with damage is able to preserve the IPL and INL thickness and retinal tissue cytostructure [7,20]. To date, GH was only tested in neuroprotective protocols, but this is the first time that GH was chronically delivered into the eye after a retinal excitotoxic damage induced by KA [7,20,21,64]. Similarly, the GH-induced recovery of retinal thickness observed in this study was mainly located in the IPL and INL. It is important to note that the IPL is mostly formed by dendritic connections between bipolar cells, amacrine cells, and retinal ganglion cells (RGCs) [65], however, in the chicken, some displaced RGCs and glia could be observed within this layer [4]. Our findings are consistent with previous reports demonstrating neurotrophic effects of GH in neurite and axonal growth [6,7,66]. It is therefore possible that the GH-induced recovery in patients with brain trauma, cognitive deficit, and spinal cord injury, at least partially includes the restoration of functional neural interconnections [6,64,67,68].

PSD95 is a post-synaptic scaffold protein associated to glutamate receptors in excitatory synapses. GH has previously been shown to increase PSD95 immunoreactivity in KA-damaged retinas, which was directly associated with its neuroprotective action [20]. Reports in mammals showed GH increased DLG4 mRNA which codes for PSD95, and this effect positively correlates with an improvement in memory [24]. We found that with DLG1, the avian homolog of DLG4 [25], gene expression is upregulated in damaged retinas chronically treated with GH, and our evidence suggests an association with the observed regenerative effect.

Neurexin-1 is a pre-synaptic transmembrane adhesion molecule expressed in excitatory synapsis and it is widely distributed in the central nervous system (CNS) [26]. In the chicken embryo, neurexin-1 mRNA is expressed in the telencephalon, optic tectum, hindbrain, and retina [27]. To our knowledge, this work shows for the first time the expression of NXRN1 in the neonatal chicken retina, and this expression was used as a marker for GH-induced regeneration. GH treatment in KA-damaged retinas strongly increased NXRN1 expression which occurs only in the presence of damage, this evidence suggests a long-term regenerative and synaptogenic effect. The regenerative effect of GH upon the IPL and INL is consistent with the localization of NXRN1 mRNA in embryonic bipolar cells and RGCs.

Recently, we showed that SNAP25, a pre-synaptic vesicle fusion protein, is strongly downregulated between 2 to 96 h after an acute KA-injury [20], however, at day 21 after KA damage, the retinal tissue upregulates SNAP25 expression. Contrary to our expectations, GH did not exert any effect upon SNAP25 expression, which is consistent with previous studies on neuroprotection [20]. Therefore, it is likely that in the chicken retina, GH is not associated with SNAP25 expression during neuroregenerative and neuroprotective processes.

GAP43 is an accepted marker for axonal growth, which is essential for the developing CNS and adult neurogenesis. Previous studies on the effects of GH against an excitotoxic injury have found that protective actions of GH reflect changes in GAP43 localization rather than relative abundance differences [20]. In addition, GH has been previously shown to increase GAP43 in a neuroretinal derived cell line (QNR/D) [19]. Similarly, GH was able to increase GAP43 immunoreactivity in damaged retinas following a post-injury administration demonstrating a role for this marker in both neuroprotection and neuroregeneration. Moreover, GAP43 inhibition has been associated with an increase of apoptotic cells after a retinal injury [29], which is consistent with GH effects increasing both GAP43 and cell survival [7,19,21].

It is well documented that GH improves cognitive function through changes in the expression of NMDA and AMPA receptors [31]. Neurocognitive functions, long-term memories, experimental LTP, and neural differentiation in rats with GH/IGF-1 hypersecretion are significantly improved [69]. Interestingly, GH administration in patients treated with methadone downregulates NMDA receptors in order to prevent excitotoxic damage [32]. Our findings demonstrate that at 21 days post-injury, NR1 (NMDA-R) and GRIK4 (KA-R) receptor subunits mRNA expression is upregulated by damage, and GH treatments in damaged retinas further increased their mRNA levels in comparison to damaged group. This suggests that glutamate receptors are involved in the retinal healing process and it is likely that GH could be working as a regenerative enhancer. This possibility is also supported by the fact that GluRs activation is not restricted to neurotransmission, since glutamate signaling is critical for cell migration, neurite growth and synapse formation [33,34,35].

In response to damage, chicken Müller cells can de-differentiate into a neural progenitor phenotype termed Müller glia-derived progenitor cells (MGPCs), which is correlated with the expression of transdifferentiation markers such as Sox2 and Pax6 [46]. The newly formed neural progenitors can proliferate but they do not differentiate into new neurons [14]. We observed that GH increased expression of Sox2 and FGF2 for at least 3 weeks. Therefore, it is likely that MGPCs extended their early phenotype stage in response to chronic GH treatment. Although we did not specifically identify Müller cells as those involved in Sox2 increase during the regenerative response observed in this study, there is a vast literature showing that changes in Sox2 correlate with the increase in neural progenitors during Müller cell transdifferentiation induced by NMDA damage [50]. In response to excitotoxic injury, an increase of Sox2 expression in the avian retina was described and it mostly occurred in both NIRG (an atypical glial cell type) and Müller/MGPCs subpopulations [14]. Considering the proportion of these cells in retinal tissue, it is likely that Sox2 changes observed in this study could be mostly related to an increase in MGPCs.

Notch signaling pathway has a critical role during the CNS development in all species and participates in the neuroregenerative process described in lower vertebrates [49,50]. Both Notch1 and Hes5 expression were analyzed and we observed that both genes were upregulated in damaged retinas treated with GH. The role of Notch signaling in neuroregeneration and neuroprotection is unclear, however, recent reports suggest that Notch signaling relies on the physiopathological context [47,51]. Some evidence suggests negative actions for Notch signaling, since the inhibition of this pathway using DAPT in hypoxic/ischemic models resulted in neuroprotection [51]. However, Notch signaling activation in the rodent retina could be associated with neuroprotection [52]. Recent results from our group suggest that an increased Notch signaling by GH is a key mediator of neuroprotection against KA-injury in the chicken retina [53].

It has been proposed that neuroprotective actions of GH are mediated by local factors such as neuropeptides, hormones, neurotrophins, and growth factors [70]. BDNF and NT3 are classical neurotrophins widely expressed in the CNS and in retinal cells [41], and both are responsive to GH stimulation in RGCs [7]. In addition, BDNF has been proposed as a therapeutic option for retinal degeneration and neuroprotection due to its strong neurotrophic actions [71]. We found that long-term GH effect after a neurotoxic insult involves BDNF gene expression, which clearly correlates with the regenerative effect observed by morphometry. There is also evidence that BMP4 and CNTF are involved in neuroprotective actions against excitotoxicity and regulate the formation of MGPCs in the chicken retina [42,43,44]. GH actions on BMP4 activity in bone development are well established [72,73] but its implications in neural function remains to be elucidated. This is the first report demonstrating an effect of GH action in BMP4 expression in retinal tissue, however, this effect was only observed in the absence of damage.

To investigate the participation of pro-survival mechanisms during the GH neuroregenerative effect, TNR-R2 and Bcl-2 were selected as molecular markers. The TNF receptor system, which includes TNF-R1 and TNF-R2, is involved in the control of life and death balance of immune cells [36], however, its function in the nervous system has not been completely demonstrated yet. TNF-R2 activity in the retina is controversial; RGC loss is increased in retinal receptor deficiency [37], but in damage-induced by ocular hypertension, there is a protective effect [38]. In this study, we observed that TNF-R2 immunoreactivity was restored to normal levels in KA-damaged retinas treated with GH. It has been conclusively demonstrated that GH induces Bcl-2 as a mediator of its anti-apoptotic effects [21,39,40], however these molecular mechanisms were reported only in neuroprotective and developmental studies. Despite the fact that GH induces a fast increase in Bcl-2 levels within 24 h [21], 5-days after the last hormone administration its immunoreactivity was higher than in damaged retinas without treatment; this result can be partially explained by the activation of complex and long-lasting paracrine interactions in this tissue system.

The endocrine dogma states that GH is a critical regulator of cell proliferation and differentiation during development, but also exerts proliferative actions in neural progenitors in vitro [74,75,76,77]. In addition, local expression of GH in the chicken embryo retina regulates developmental apoptosis, cell differentiation, and proliferation [7,17]. In this study, we found that GH mRNA expression was strongly increased by KA-damage, which is consistent with previous studies in the green iguana retina [78], although this autocrine/paracrine interaction was not apparently enough to prevent damage progression or to significantly regenerate the retinal structure. It is possible that, in response to injury, local GH participates in the regulation of MGPCs proliferation and in neurite regeneration. We determined proliferation though PCNA mRNA expression at day 21 post-injury but there was no significant effect induced by KA or KA + GH. This lack of proliferation 3 weeks after damage is consistent with the literature, since the proliferative stage shows a significative decrease at day 14 post-injury [14]. Considering our PCNA data, we decided to look for proliferation at a shorter time, since it has been described that at 8 days post injury, damaged retinas showed a high proliferation rate [14]. Thus, we used Brd-U staining to analyze this issue and found a strong proliferative effect induced by KA at day 8, and this effect was further increased with GH treatments in damaged retinas. The retinal cells with proliferative activity after a neurotoxic damage are well characterized as MGPCs [14]; however, it is possible that GH actions in proliferation could also include a low proportion of other different cell types.

In conclusion, this work provides evidence that chronic GH treatment enhanced regeneration in the retinal epithelium when applied after an acute neurotoxic injury using a glutamate receptor agonist. Results summarized in Table 4 indicate that GH potentiates retinal cell proliferation and significantly upregulates markers associated with synaptic restoration, neurite growth, and pro-survival effects, which may be relevant to recover neural connectivity after injury. It is important to note that GH actions are conditioned by damage, which are not observed in healthy retinal tissue. This work provides additional evidence about the therapeutic potential of GH as a neurotrophic factor, which deserves further investigation.

## Figures and Tables

**Table 1:** Factors associated to regeneration markers in the avian retina.

Name	Description	Retinal Marker	References
Discs large homolog 1 (DLG1)	Post-synaptic scaffold protein (avian PSD95)	Synaptogenic	[24,25]
Neurexin 1 (NRXN1)	Synaptic cell-surface protein	Synaptogenic	[26,27]
Synaptosomal nerve-associated protein 25 (SNAP25)	Synaptic vesicle fusion protein	Synaptogenic	[20,28]
Growth associated protein 43 (GAP43)	Expressed during axonal growth regulation	Axogenic	[29,30]
NR1	NMDA-R subunit	Synaptogenic	[31,32,33,34]
GRIK4	KA-R subunit	Synaptogenic	[33,35]
Tumor necrosis factor receptor 2 (TNF-R2)	Pro-survival receptor	Neurotrophic	[36,37,38]
B-cell lymphoma 2 (Bcl-2)	Anti-apoptotic protein	Anti-apoptotic	[21,39,40]
Brain-derived neurotrophic factor (BDNF)	Neurotrophic factor	Neurotrophic	[7,41]
Bone morphogenic protein 4 (BMP4)	Pro-survival growth factor	Neurotrophic	[42,43,44]
Fibroblast growth factor (FGF2)	Proliferative growth factor	Proliferation and transdifferentiation	[13,45]
Sex determining Region Y-box 2 (Sox2)	Pluripotency transcription factor	Transdifferentiation	[14,46]
Achaete-scute homolog 1 (Ascl1)	Transcription factor associated to differentiation	Transdifferentiation	[47,48]
Notch Receptor 1 (Notch1)	Membrane receptor—Notch signaling element	Transdifferentiation	[47,49,50,51,52,53]
Hes5	Responsive gene—Notch signaling pathway	Transdifferentiation	[49,50,51,52,53]
Proliferating cell nuclear antigen (PCNA)	Nuclear proliferative protein	Proliferation	[54,55]

**Table 2:** Oligonucleotides.

Target	Primer	Sequence (5′-3′)	Size	Accesion #
cNotch1	Fwd	GGCTGGTTATCATGGAGTTA	155 bp	NM_001030295.1
	Rev	CATCCACATTGATCTCACAG		
cHes5	Fwd	GGAGAAGGAGTTCCAGAGAC	171 bp	NM_001012695.1
	Rev	ATTTGCAGAGCTTCTTTGAG		
cBDNF	Fwd	AGCAGTCAAGTGCCTTTGGA	167 bp	NM_001031616
	Rev	TCCGCTGCTGTTACCCACTCG		
cASCL	Fwd	AGGGAACCACGTTTATGCAG	188 bp	NM_204412.1
	Rev	TTATACAGGGCCTGGTGAGC		
cSox2	Fwd	AGGCTATGGGATGATGCAAG	163 bp	NM_205188.2
	Rev	GTAGGTAGGCGATCCGTTCA		
cFGF2	Fwd	TGCAGCTTCAAGCAGAAGAA	173 bp	NM_205433.1
	Rev	CTTCCGTGACCGGTAAGTGT		
cDLG1	Fwd	ACCAGCCAGAAGAGATCCCT	162 bp	XM_025153616.1
	Rev	TGGAGTTACCTGCCGTGCTT		
cBMP4	Fwd	CGCTGGGAGACCTTTGATGT	153 bp	NM_205237.3
	Rev	CCCCTGAGGTAAAGATCGGC		
cSNAP25	Fwd	GCCTGCCCGTGTGGTAGAT	185 bp	NM_205458.1
	Rev	TCTGGCGATTCTGTGTGTCG		
cNRXN1	Fwd	CCACTCTGATCATTGACCGGG	392 bp	NM_001198975.1
	Rev	CGCCAGACCTTCCACATAGT		
c18 s	Fwd	CTCTTTCTCGATTCCGTGGGT	100 bp	M59389
	Rev	TTAGCATGCCAGAGTCTCGT		
cNR1	Fwd	ACGGTCCCACCATACTCTCA	156 bp	NM_206979.1
	Rev	AGCCTTGGACTCTCTCTCCT		
cGH	Fwd	CGCACCTATATTCCGGAGGAC	128 bp	NM_204359
	Rev	GGCAGCTCCATGTCTGACT		
cGRIK4	Fwd	AAATGTCCCAAGGAGGAGGACCAC	215 bp	XM_015298177.2
	Rev	AAACTGTCCTTGCAGAGGATGATG TTGCGC		

**Table 3:** Antibodies.

Target	Host/Type	Dilution	Source	Cat.No
Bcl-2	mouse/polyclonal	1:1000	Invitrogen	138800
β-Actin	mouse/monoclonal	1:5000	Santa cruz	SC-47778
TNF-R2	goat/polyclonal	1:1000	Santa Cruz	SC-1072
GAP 43	Mouse/monoclonal	1:2000	Sigma-Aldrich	GAP-7B10
BrdU	Rat/monoclonal	1:100	Abcam	AB-6326
Mouse IgG	Goat/HRP conjugated	1:6000	Abcam	AB-20043
Rat IgG	Goat/ Alexa fluor 488	1:1000	Abcam	AB-150157
Goat IgG	Rabbit/HRP	1:6000	Life tech.	811620

**Table 4:** Effect of growth hormone (GH) upon the expression of retinal markers in regenerative response after kainic acid (KA)-induced damage.

		Response per Treatment			
		GH vs Sham	KA vs Sham	KA + GH vs Sham	KA + GH vs KA
Synaptogenic	DLG1	-	-	**↑↑**	-
	NRXN1	-	-	**↑↑↑↑**	**↑↑**
	SNAP25	-	**↑↑**	-	-
	GAP43	-	-	**↑**	-
	NR1	-	**↑↑↑↑**	**↑↑↑↑**	**↑↑↑↑**
	GRIK4	↓	**↑**	**↑↑↑↑**	**↑↑↑**
Survival	TNF-R2	-	↓↓	-	-
	Bcl-2	-	-	-	**↑**
	BDNF	-	-	**↑↑↑**	-
	BMP4	**↑**	-	-	-
Transdifferentiation	FGF2	-	-	**↑**	**↑↑**
	Sox2	-	-	**↑↑**	**↑**
	Ascl1	-	-	-	-
	Notch1	-	-	-	**↑↑↑**
	Hes5	-	↓↓	**↑↑↑**	**↑↑↑↑**
	PCNA	-	-	-	-

Upregulation: **↑** (*p* < 0.05); **↑↑** (*p* < 0.01); **↑↑↑** (*p* < 0.001); **↑↑↑↑** (*p* < 0.0001); Downregulation: **↓** (*p* < 0.05); **↓↓** (*p* < 0.01); **-** No effect.
